# Association of short sleep duration and trabecular bone score

**DOI:** 10.1038/s41598-021-99410-w

**Published:** 2021-10-06

**Authors:** Yi-Chih Shiao, Wan-Ting Chen, Wei-Liang Chen

**Affiliations:** 1 Division of Family Medicine, Department of Family and Community Medicine, Tri-Service General Hospital, Taipei, Taiwan, Republic of China; 2 School of Medicine, National Defense Medical Center, Taipei, Taiwan, Republic of China; 3 Division of Geriatric Medicine, Department of Family and Community Medicine, Tri-Service General Hospital, National Defense Medical Center, Number 325, Section 2, Chang-gong Rd, Nei-Hu District, 114, Taipei, Taiwan, Republic of China

**Keywords:** Diagnostic markers, Prognostic markers, Endocrinology

## Abstract

Short sleep duration has been found to be associated with bone health deterioration by using bone mineral density (BMD). Only a few attempts have been made to assess the association of sleep duration and bone by utilizing the trabecular bone score (TBS). The aim of this study was to examine the association between sleep duration and TBS from a national database. A total of 4480 eligible participants older than 20 years who attended the United States National Health and Nutrition Examination Survey (NHANES) from 2005 to 2006 with TBS data and self-reported sleep duration. The association between sleep duration and TBS was investigated using a multivariate regression model with covariate adjustment. TBS was lowest in individuals with a short sleep duration (≤ 5 h) and it was increased in those with longer self-reported total sleep times. After a full adjustment for covariates, those sleeping less than 5 h had a significantly lower TBS than the reference group (sleep duration of 7 h). In subgroup analyses, an association between short sleep duration (≤ 5 h) and lower TBS persisted in older ages (≥ 60 years old), women, obese adults (BMI ≥ 30 kg/m^2^), and non-Hispanic Whites. Short sleep duration is associated with low TBS in women, obese adults (BMI ≥ 30 kg/m^2^), and non-Hispanic whites. Strict self-monitoring of body weight, well-tailored controls of underlying disease(s), and adequate sleep may help prevent osteoporosis.

## Introduction

Osteoporosis is a systemic skeletal disorder, and it has been found to be strongly associated with short sleep duration^1,2^. The root cause of short sleep-duration-related-bone deterioration may derive from the disruption of bone turnover marker (BTM) rhythmicity^3^ on a molecular level as a result of dyssynchronization of the central-peripheral bone clock^4^. The standard method of diagnosing osteoporosis is with dual-energy X-ray absorptiometry (DXA) for measuring BMD^5^, implemented since 1994^6^.

BMD is part of the fracture risk assessment tool (FRAX) and it has been applied to predict the 10-year probability of a major osteoporotic fracture^7^. Nevertheless, evaluation of fracture risk by using BMD alone could be insufficient for vulnerable populations with normal or higher bone density levels^8^. Degenerative changes, such as osteoarthrosis, scoliosis, and vertebral compression fracture may result in overestimation of lumbar BMD^9,10^. In contrast, the TBS is less influenced by vertebral fractures^11^. Current evidence suggests that BMD in combination with TBS could predict major osteoporotic fracture more accurately in elderly individuals than BMD alone^12^.

TBS, an indirect measurement of gray-level texture derived from lumbar spine DXA, is a parameter that estimates bone quality by evaluating the trabecular microarchitecture^13,14^. TBS helps detect individuals with degraded microarchitecture but normal BMD^15^ and accurately predict osteoporotic fractures in several studies that are independent of areal BMD and other clinical risk factors^16,17^. TBS can be an advantageous tool for obtaining more comprehensive bone data in comparison to standard BMD measurements^13,18^. TBS has been utilized in a few of sleep medicine studies. Nimitphong et al. explored the association between severities of obstructive sleep apnea and TBS in 81 individuals with type 2 diabetes mellitus^19^. However, the generalizability of this article is limited by the characteristics of the target participants. The relationship between sleep duration and bone health assessed by TBS among a large sample of people is still unclear.

There is a paucity of research examining the association between sleep duration and TBS. To address this, we investigated the relationship between sleep duration and TBS by examining data from the NHANES from 2005 to 2006. We hypothesized that extremely short sleep durations (≤ 5 h) would be associated with decreased TBS.

## Results

### Characteristics and demographic data of the participants

Table 1 demonstrates both the demographic and clinical characteristics of the individuals categorized by separate sleep durations. The mean age was 48.36 ± 18.91 years, and men comprise 48% of the participants. Men predominated (50.8%) in the ≤ 5-h sleep duration group but this proportion decreased as sleep durations were prolonged (49.3% in the 6-h group, 48.8% in the 7-h group, 47.3% in the 8-h group, and 38.9% in the ≥ 9-h group), whereas women made up increasingly higher percentages in the categories of a longer sleep duration. In the ≤ 5 h sleep duration group, individuals had a significantly lower level of TBS (1.3530 ± 0.15188, *P* = 0.001) and a higher body mass index (BMI) (30.19 ± 7.59, *P* < 0.001) than the other groups.

**Table 1:** Characteristics of study participants.

Characteristic	Sleep duration at night						
	≦5 h N = 677	6 h N = 1008	7 h N = 1190	8 h N = 1240	≧9 h N = 365	Total N = 4480	*P*-value
Continuous variables*							
Age (years)**	47.90(17.91)	48.12(17.95)	46.97(17.75)	49.44(20.00)	50.81(22.46)	48.36(18.91)	0.002
BMI (kg m^−2^)***	30.19(7.59)	29.14(6.76)	28.23(5.79)	28.34(6.40)	28.58(8.66)	28.79(6.76)	< 0.001
CRP (mg/dL)**	0.51(0.70)	0.48(0.84)	0.42(0.86)	0.51(0.88)	0.59(0.89)	0.49(0.84)	0.008
Fasting Glucose (mg/dL)	106.11(34.85)	105.07(35.09)	101.67(22.52)	105.96(36.63)	107.44(40.93)	104.84(33.40)	0.124
Total spine BMD	1.0452(0.15425)	1.0460(0.15749)	1.0395(0.14507)	1.0413(0.15007)	1.0199(0.16346)	1.0409(0.15215)	0.222
TBS**	1.3530(0.15188)	1.3680(0.14736)	1.3851(0.13486)	1.3740(0.13972)	1.3609(0.14900)	1.3716(0.14311)	0.001
**Categorical variables**^**a**^							
Male**	344(50.8)	497(49.3)	581(48.8)	586(47.3)	142(38.9)	2150(48.0)	0.004
Race-ethnicity***							< 0.001
Mexican American	124(18.3)	196(19.4)	238(20.0)	282(22.7)	69(18.9)	909(20.3)	
Other Hispanic	21(3.1)	32(3.2)	46(3.9)	33(2.7)	6(1.6)	138(3.1)	
Non-Hispanic White	258(38.1)	448(44.4)	666(56.0)	682(55.0)	202(55.3)	2256(50.4)	
Non-Hispanic Black	246(36.3)	288(28.6)	193(16.2)	202(16.3)	72(19.7)	1001(22.3)	
Snorting/ stop breathing ***	160(26.6)	224(24.3)	189(17.1)	194(17.2)	56(17.0)	823(20.2)	< 0.001
Coronary heart disease	33(4.9)	43(4.3)	28(2.4)	58(4.7)	13(3.6)	175(3.9)	0.056
Cancer/Malignancy**	47(6.9)	68(6.7)	99(8.3)	111(9.0)	46(12.6)	371(8.3)	0.001
Smoking	341(50.4)	488(48.4)	542(45.5)	579(46.7)	171(46.8)	2121(47.3)	0.464

Continuous variables are presented as mean (standard deviation). Categorical variables are presented as number (percentage).

*BMD* bone mineral density, *BMI* body mass index, *CRP* C-reactive protein, *TBS* Trabecular bone score.

Asterisks indicate statistical significance: *P < 0.05, **P < 0.01, ***P < 0.001.

### Short sleep duration is associated with a decreased TBS

The results from the regression analysis between sleep duration and TBS are shown in Table 2. Participants in the ≤ 5-h sleep duration group had a lower level of TBS than the reference group. The unadjusted regression coefficients were − 0.025 (*P* < 0.001), − 0.014 (*P* = 0.009), − 0.008 (*P* = 0.092), and − 0.019 (*P* = 0.013) for groups with sleep durations of ≤ 5 h, 6 h, 8 h, and ≥ 9 h, respectively, compared with that of the reference group with a 7 -hour sleep duration. The result remained statistically significant with a lower level of TBS in the group with the shortest sleep duration (≤ 5 h) after full adjustment for multiple variables.

**Table 2:** Association between different sleep duration and trabecular bone score.

Sleep duration^a^	Unadjusted model		Totally adjusted model	
	β^b^ (95% CI)	*P* value	β^b^ (95% CI)	*P* value
≦5 h	− 0.025 (− 0.037, − 0.014)	< 0.001***	− 0.018 (− 0.033, − 0.003)	0.018*
6 h	− 0.014 (− 0.024, − 0.003)	0.009**	− 0.004 (− 0.018, 0.010)	0.573
7 h	Reference	Reference	Reference	Reference
8 h	− 0.008 (− 0.018, 0.001)	0.092	0.011 (− 0.003, 0.024)	0.113
≧9 h	− 0.019 (− 0.034, − 0.004)	0.013*	0.006 (− 0.013, 0.026)	0.526

Adjusted for age, race/ethnicity, gender, fasting glucose, C reactive protein, smoke, coronary heart disease, cancer, snorting, total spine bone mineral density (BMD).

*CI* confidence interval.

^a^Length were the reference group.

^b^β coefficient can be interpreted as differences in the mean TBS comparing subjects in the other 4 groups of sleep duration to those in the 7-h sleep length.

Asterisks indicate statistical significance: *P < 0.05, **P < 0.01, ***P < 0.001.

### Association of sleep duration and TBS after stratification by age, sex, BMI, and ethnicity

The results of the linear and logistic regression models adjusted for various confounders and separated by age are presented in Table 3. In the unadjusted model, participants with aged < 60 years in the ≤ 5 -hour sleep duration group (β = − 0.021, *P* = 0.001) and 6-h sleep duration group (β = − 0.012, *P* = 0.038) showed negative associations between shorter sleep duration and TBS compared with the reference group. Participants aged ≥ 60 in the ≤ 5 -hour sleep duration group (β = − 0.043, *P* < 0.001) showed a negative association between a short sleep duration and TBS compared with the reference group in the unadjusted model. After full adjustment, there was only one significantly negative association between a short sleep duration and TBS among participants aged ≥ 60 in the ≤ 5 -hour sleep duration group (β = − 0.054, *P* = 0.006) compared with the reference group.

**Table 3:** Age-specific association between sleep duration and trabecular bone score (TBS).

Sleep duration^a^	Unadjusted model				Totally adjusted model			
	Age < 60		Age≧ 60		Age < 60		Age≧ 60	
	β^b^ (95% CI)	*P* value	β^b^ (95% CI)	*P* value	β^b^ (95% CI)	*P* value	β^b^ (95% CI)	*P* value
**≦**5 h	− 0.021 (− 0.033, − 0.009)	0.001*	− 0.043 (− 0.065, − 0.021)	< 0.001***	− 0.013 (− 0.029, 0.003)	0.115	− 0.054 (− 0.092, − 0.016)	0.006**
6 h	− 0.012 (− 0.023, − 0.001)	0.038*	− 0.014 (− 0.032, 0.005)	0.143	− 0.007 (− 0.022, 0.008)	0.373	0.008 (− 0.024, 0.040)	0.634
7 h	Reference	Reference	Reference	Reference	Reference	reference	reference	reference
8 h	0.008 (− 0.003, 0.019)	0.165	− 0.008 (− 0.025, 0.009)	0.341	0.010 (− 0.005, 0.025)	0.205	0.011 (− 0.017, 0.039)	0.442
**≧**9 h	− 0.001 (− 0.019, 0.017)	0.934	− 0.011 (− 0.034, 0.012)	0.363	0.006 (− 0.017, 0.029)	0.595	− 0.006 (− 0.043, 0.032)	0.756

Adjusted for age, race/ethnicity, gender, fasting glucose, C reactive protein, smoke, coronary heart disease, cancer, snorting, total spine bone mineral density (BMD).

*CI* confidence interval.

^a^Length were the reference group.

^b^β coefficient can be interpreted as differences in the mean TBS comparing subjects in the other 4 groups of sleep duration to those in the 7-h sleep length.

Asterisks indicate statistical significance: *P < 0.05, **P < 0.01, ***P < 0.001.

After the stratification of the associations by sex, the results of the linear and logistic regression models adjusted for several confounding factors are presented in Table 4. In the unadjusted model, women, not men, demonstrated an association (β = − 0.048, *P* < 0.001) between a ≤ 5-h sleep duration and lower TBS compared with the reference group. After full adjustment, this result remained statistically significant (β = − 0.023, *P* = 0.039) with lower TBS in the shortest sleep duration (≤ 5 h).

**Table 4:** Gender-specific association between sleep duration and trabecular bone score (TBS).

Sleep duration^a^	Unadjusted model				Totally adjusted model			
	Men		Women		Men		Women	
	β^b^ (95% CI)	*P* value	β^b^ (95% CI)	*P* value	β^b^ (95% CI)	*P* value	β^b^ (95% CI)	*P* value
**≦**5 h	− 0.006 (− 0.022, 0.010)	0.467	− 0.048 (− 0.065, − 0.031)	< 0.001***	− 0.011 (− 0.032, 0.010)	0.307	− 0.023 (− 0.044, − 0.001)	0.039*
6 h	− 0.010 (− 0.024, 0.004)	0.158	− 0.017 (− 0.032, − 0.002)	0.022*	− 0.004 (− 0.024, 0.016)	0.696	− 0.003 (− 0.021, 0.016)	0.769
7 h	Reference	Reference	Reference	Reference	Reference	Reference	Reference	Reference
8 h	− 0.003 (− 0.016, 0.011)	0.703	− 0.015 (− 0.029, − 0.001)	0.041*	0.013 (− 0.006, 0.033)	0.181	0.010 (− 0.008, 0.028)	0.290
**≧**9 h	− 0.025 (− 0.047, − 0.003)	0.023*	− 0.015 (− 0.035, 0.006)	0.169	− 0.015 (− 0.045, 0.015)	0.319	0.028 (0.004, 0.053)	0.024*

Adjusted for age, race/ethnicity, gender, fasting glucose, C reactive protein, smoke, coronary heart disease, cancer, snorting, total spine bone mineral density (BMD).

*CI* confidence interval.

^a^Length were the reference group.

^b^β coefficient can be interpreted as differences in the mean TBS comparing subjects in the other 4 groups of sleep duration to those in the 7-h sleep length.

Asterisks indicate statistical significance: *P < 0.05, **P < 0.01, ***P < 0.001.

The results of the linear/logistic regression model after adjustment for several confounders and categorization by BMI (≥ 30 versus < 30 kg/m^2^) are presented in Table 5. In the BMI ≥ 30 group, unadjusted regression coefficients were − 0.032 (*P* = 0.005), − 0.023 (*P* = 0.024), − 0.026 (*P* = 0.012), and − 0.042 (*P* = 0.007) for groups with sleep durations of ≤ 5 h, 6 h, 8 h, and ≥ 9 h, respectively, compared with those of the reference group of a 7 -hour sleep duration. In the fully adjusted model, the BMI ≥ 30 group demonstrated a significantly association (β = − 0.029, *P* = 0.049) between the shortest sleep duration (≤ 5 h) and TBS, consistent with the results in the unadjusted model.

**Table 5:** BMI specific association between sleep duration and trabecular bone score (TBS).

Sleep duration^a^	Unadjusted model				Totally adjusted model			
	BMI < 30		BMI ≥ 30		BMI < 30		BMI ≥ 30	
	β^b^ (95% CI)	*P* value	β^b^ (95% CI)	*P* value	β^b^ (95% CI)	*P* value	β^b^ (95% CI)	*P* value
≦5 h	− 0.011 (− 0.022, 0.000)	0.046*	− 0.032 (− 0.053, − 0.010)	0.005**	− 0.007 (− 0.018, 0.004)	0.241	− 0.029 (− 0.057, 0.000)	0.049*
6 h	− 0.004 (− 0.013, 0.006)	0.427	− 0.023 (− 0.043, − 0.003)	0.024*	0.001 (− 0.009, 0.011)	0.862	− 0.007 (− 0.034, 0.020)	0.606
7 h	Reference	0.467	Reference	Reference	Reference	Reference	Reference	Reference
8 h	− 0.003 (− 0.012, 0.006)	0.135	− 0.026 (− 0.045, − 0.006)	0.012*	0.000 (− 0.009, 0.010)	0.987	0.012 (− 0.015, 0.040)	0.378
≧9 h	− 0.010 (− 0.024, 0.003)	Reference	− 0.042 (− 0.072, − 0.011)	0.007**	0.003 (− 0.010, 0.017)	0.621	− 0.008 (− 0.048, 0.032)	0.692

Adjusted for age, race/ethnicity, gender, fasting glucose, C reactive protein, smoke, coronary heart disease, cancer, snorting, total spine bone mineral density (BMD).

*BMI* body mass index, *CI* confidence interval.

^a^Length were the reference group.

^b^β coefficient can be interpreted as differences in the mean TBS comparing subjects in the other 4 groups of sleep duration to those in the 7-h sleep length.

Asterisks indicate statistical significance: *P < 0.05, **P < 0.01, ***P < 0.001.

We also used linear and logistic regression models to adjust the confounding variables to evaluate the ethnicity-specific association between sleep duration and TBS, as shown in Table 6. There was statistical significance in 2 associations after full adjustment. One is an inverse association between the shortest sleep duration (≤ 5 h) and TBS in the non-Hispanic white group (β = − 0.024, *P* = 0.041), and the other is a positive association between a slightly longer sleep duration (8 h) and TBS in the other Hispanic group (β = 0.073, *P* = 0.045). There was no statistical significance among Mexican Americans, non-Hispanic black individuals, or other races before and after full adjustment.

**Table 6:** Ethnicity specific association between sleep duration and trabecular bone score (TBS).

Sleep duration^a^	Unadjusted model				
	Mexican American	Other Hispanic	Non-Hispanic White	Non-Hispanic Black	Other Races
	β^b^ (95% CI)	β^b^ (95% CI)	β^b^ (95% CI)	β^b^ (95% CI)	β^b^ (95% CI)
≦5 h	0.001 (− 0.024, 0.026)	− 0.045 (− 0.100, 0.010)	− 0.040 (− 0.058, − 0.023)***	− 0.017 (− 0.041, 0.007)	− 0.025 (− 0.082, 0.032)
6 h	− 0.005 (− 0.027, 0.017)	0.035 (− 0.012, 0.081)	− 0.022 (− 0.036, − 0.007)**	− 0.007 (− 0.031, 0.016)	− 0.019 (− 0.067, 0.029)
7 h	Reference	Reference	Reference	Reference	Reference
8 h	− 0.003 (− 0.024, 0.017)	0.058 (0.09, 0.107)*	− 0.014 (− 0.028, − 0.001)*	− 0.013 (− 0.038, 0.013)	0.029 (− 0.021, 0.079)
≧9 h	− 0.020 (− 0.056, 0.015)	− 0.063 (− 0.142, 0.016)	− 0.014 (− 0.034, 0.006)	− 0.025 (− 0.061, 0.011)	− 0.023 (− 0.100, 0.054)

Sleep duration^a^	Totally adjusted model				
	Mexican American	Other Hispanic	Non-Hispanic White	Non-Hispanic Black	Other Races
	β^b^ (95% CI)	β^b^ (95% CI)	β^b^ (95% CI)	β^b^ (95% CI)	β^b^ (95% CI)
≦5 h	0.006 (− 0.027, 0.039)	0.014 (− 0.067, 0.096)	− 0.024 (− 0.047, − 0.001)*	− 0.021 (− 0.050, 0.008)	0.038 (− 0.060, 0.137)
6 hours6 hours	− 0.005 (− 0.035, 0.025)	0.037 (− 0.028, 0.102)	− 0.002 (− 0.022, 0.017)	− 0.005 (− 0.035, 0.025)	0.005 (− 0.061, 0.072)
7 h	Reference	Reference	Reference	Reference	Reference
8 h	0.018 (− 0.009, 0.045)	0.073 (0.002, 0.144)*	0.012 (− 0.006, 0.031)	− 0.006 (− 0.038, 0.026)	0.032 (− 0.044, 0.108)
≧9 h	0.014 (− 0.032, 0.059)	0.028 (− 0.087, 0.142)	0.004 (− 0.022, 0.030)	− 0.014 (− 0.058, 0.030)	0.069 (− 0.022, 0.161)

Adjusted for age, race/ethnicity, gender, fasting glucose, C reactive protein, smoke, coronary heart disease, cancer, snorting, total spine bone mineral density (BMD).

*BMI* body mass index, *CI* confidence interval.

^a^Length were the reference group.

^b^β coefficient can be interpreted as differences in the mean TBS comparing subjects in the other 4 groups of sleep duration to those in the 7-h sleep length.

Asterisks indicate statistical significance: *P < 0.05, **P < 0.01, ***P < 0.001.

## Discussion

In our study, we investigated the association between self-reported sleep duration and bone health assessed by TBS among a large sample of the United States population older than 20 years old. Remarkably, our results show that the association between short sleep duration (equal to or less than 5 h) and lower TBS is closely related to older ages (≥ 60 years old), women, obese individuals with BMI equal to or more than 30 kg/m^2^, and only non-Hispanic Whites after full adjustment.

A growing amount of evidence validates the importance of adequate sleep. From the perspective of neuroscience, malregulation of the sympathetic autonomic nervous system will cause a loss of cortical bone thickness^2^. Bone loss occurs as a result of alterations in the circadian system and subsequent disruption of bone turnover markers^4^. From another point of view, sleep can have a great influence on bone health through hormone regulation such as leptin^2,20^ and growth hormone^20^. A relationship between sleep duration and bone health is seen in animals as well. Sleep restriction and long-term inadequate sleep lead to reduced bone formation and bone resorption-related markers in rats^21,22^. To mitigate adverse impacts of short sleep duration on bone health, sleeping more than 7 h per day is recommended by the National Sleep Foundation^23^.

A wealth of evidence strongly suggests that age and sex must be taken into consideration in regard to bone health. Aging-related bone loss is a complicated entity and is mainly due to changes in the composition of the bone marrow with subsequent increases in adipocytes and functional declines in osteoblasts^24^. With advancing age, a reduction in TBS occurs in both male and female individuals^25^. In line with our findings, Ochs-Balcom HM et al. reported that short sleep duration (equal to or less than 5 h) was closely related to lower BMD among 11,084 postmenopausal women^1^. In clinical settings, an appropriate combination of patient characteristics and histories would facilitate an accurate evaluation of bone health^26^.

Reviewing recent studies, a wide range of conditions would be linked to reduced TBS. Obesity has been reported to be related to decreased TBS^27,28^ and short sleep duration^29,30^. Patients with impaired fasting glucose (IFG) have lower TBS than those with normal glucose levels^31^. Furthermore, once diagnosed with diabetes, patients have reduced TBS independent of BMD and they have a higher fracture risk^31^. In a cohort of 2758 people, TBS was lower while BMD was higher in diabetic patients than in those without diabetes and it had an inverse association with insulin resistance in a homeostasis model assessment^32^. This implies that TBS could be utilized in risk evaluation in addition to BMD and facilitate clinicians to encourage control of blood glucose levels to prevent skeletal fragility in diabetes. Due to the above conditions, we carefully adjusted for BMD with biomarkers of the common metabolic syndrome, and the association remained unchanged after the adjustments.

It remained statistical significance between TBS and short sleep duration after we adjusted for common comorbidities. Reduced TBS has been shown in current or ever smokers with or without chronic obstructive pulmonary disease^33^. Similar findings are seen in patients with cancer. Patients with differentiated thyroid cancer with thyroid-stimulating hormone (TSH) suppressive and nonsuppressive therapies have been followed by TBS, and the results shows that postmenopausal participants in both therapies have lower TBS unrelated to the levels of TSH^34^. Furthermore, this research team found that the rate of diagnosis of osteoporosis was 16.1%, while the prevalence of reduced TBS was 54.7%^34^. Osteoporosis might be underdiagnosed because it is diagnosed by BMD only. This reminds us of the potential for osteoporosis within a wide spectrum of diseases from benign to malignant.

Furthermore, our subgroup analyses revealed that associations between TBS and sleep duration were specific to certain races. Among the different groups, only non-Hispanic whites presented a significant association between shortest sleep duration (≤ 5 h) and decreased TBS in the fully adjusted model. The evidence for an association between sleep duration and TBS by race/ethnicity is limited. One study in 2017 reported that TBS varies by race/ethnicity^35^. Further research is necessary and a TBS database including race information should be established.

The strengths of our research include the use of a survey with a large sample size at the nationwide level and the use of a model with multivariable adjustment to control for relevant confounding factors. In the subgroup analyses, groups characterized by age, sex, obesity, and race were evaluated in a cautious manner. Our data could help establish an appropriate strategy for improving sleep duration and preventing osteoporosis. Education about sufficient sleep is recommended for a wide range of patients and early identification of these potential frailty groups facilitates our management with medications and advanced comprehensive strategies of care in the future. Based on the current understanding of sleep, advocating enough sleep should be integrated into patient education in clinical practice.

Nonetheless, this study has several limitations. The vague definitions of precise sleep components in our study are due to our methodology. To fix these limitations, an appropriate method with a fitted questionnaire may help future research teams clarify contributions from different parts of sleep with varying degrees of impact on bone health. In addition, our findings could not represent the entire population on the basis of differences in racial or genetic characteristics, baseline TBS, nutritional support, and environment. Hence, our results obtained for TBS should be carefully interpreted.

## Conclusion

Positive associations between sleep duration and TBS are observed in the U.S. adult population. Our findings provide a better understanding of the associations between sleep duration and TBS. Additional studies should be conducted based on the current data to elucidate the mechanism behind this relationship.

## Methods

### Ethics statement

Use of the dataset from the NHANES was approved by the National Center for Health Statistics (NCHS) Institutional Review Board (IRB) in compliance with the revised Declaration of Helsinki. All informed consent was obtained prior to data collection because this study was a retrospective cross-sectional study with minimal human subject contact and privacy risks.

### Study populations

The individuals in this study, 4,480 participants in total, were derived from the 2005–2006 NHANES dataset^36,37^, a national survey designed by the National Center for Health Statistics of the Centers for Diseases Control and Prevention (CDC). NHANES is a continuing series of cross-sectional, multistage population-based surveys to estimate the health and nutritional conditions of the United States civilian and noninstitutionalized populations. It has been published on the NHANES authorized website since 1999 with open access to download and analyze the data. Large-scale household interviews, physical examinations, and laboratory data were collected from the NHANES database.

### Measurement of BMD

All images of total spine BMD scans were acquired with Hologic QDR-4500A fan-beam densitometer (Hologic, Inc., Bedford, Massachusetts) processing by using Hologic software, version 8.26:a3*. The exclusion criteria included individuals with a positive urine pregnancy test and/or a self-reported history of radiographic contrast material (barium) use in the past 7 days or without complete information about the imaging results or participant refusal.

### Measurement of the level of TBS

TBS is a derivative of BMD scans that assesses the trabecular microarchitecture by estimating squared gray-level differences in the pixels of an anterior–posterior (AP) DXA scan^13,38^. All TBS scans were acquired by Hologic APEX v3.0 software with Hologic QDR-4500A fan-beam densitometers (Hologic, Inc., Bedford, Massachusetts). Subsequent data processing was performed via TBS software (Med-Imap SA TBS Calculator version 2.1.0.2). The exclusion criteria were the same as those in the aforementioned BMD measurement protocol.

### Measurement of sleep duration

Sleep duration was evaluated carefully with the survey item, “How much sleep {do you/does SP} usually get at night on weekdays or workdays?” Answers were recorded in hours and in whole numbers by the participants. People with a sleep duration of 7 h were defined as a reference group due to its promotion of desirable health as part of consensus standards^39^, and the others were categorized into different classes from short to long, including ≤ 5, 6, 8, and ≥ 9 h, to reach the optimal cutoff value of the measurement to predict the risk.

### Assessment of covariates

The associated information concerning variables, such as age, sex, race/ethnicity (Mexican American, other Hispanic, non-Hispanic white, and non-Hispanic black), BMI, use of tobacco, snorting/stop breathing, and medical conditions diagnosed by doctors, including coronary heart disease and cancer/malignancy, were collected by self-report. BMI was calculated as measured weight in kilograms divided by the square of height in meters. Smoking status was assessed by the item, “Smoked at least 100 cigarettes in life.” Snorting or stop breathing was assessed with the item, “How often have you snorted/stopped breathing in the past 12 months?” The answers were distinguished as either a clinical problem (1–2 nights/week to 5 or more nights/week) or not.

C-reactive protein (CRP) was quantified with latex-enhanced nephelometry (Dade Behring Inc, United States). Fasting glucose was measured by the hexokinase enzymatic method according to Roche Hitachi 911 analyzers. We applied standardized methods with certificates of accuracy according to the CDC guidelines for all calculations and protocols.

### Statistical analysis

The chi-square test for trend was used in the analysis of descriptive data. A 7 -hour sleep duration was recommended in adults (26 ~ 64 years old) and older adults (≥ 65 years old) by the National Sleep Foundation based on a previous study^23^. The association between sleep duration and TBS was estimated by multivariate linear regression with TBS as the dependent, continuous variable. This model was fully adjusted for age, race/ethnicity, BMI, CRP, fasting glucose, total spine BMD, snorting/stopped breathing, coronary heart disease, cancer, and smoking, and was used to control numerous confounders in the determination of the association between sleep duration classes and TBS. Furthermore, we evaluated the association between sleep duration and TBS with the use of multivariate linear and logistic regression among enrolled participants by age (< 60, ≥ 60-year-old), sex (male, female), BMI (< 30, ≥ 30 kg/m2), and ethnicity.

All data were analyzed with Statistical Product and Service Solutions version 18.0 for Windows (SPSS Inc., Chicago, Illinois, United States). A two-sided *P* < 0.05 was considered statistically significant.
